# Diagnóstico de Fibrilação Atrial na Comunidade Utilizando Eletrocardiograma e Autorrelato: Análise Transversal do ELSA-Brasil

**DOI:** 10.36660/abc.20190873

**Published:** 2021-09-01

**Authors:** Itamar S. Santos, Paulo A. Lotufo, Luisa Brant, Marcelo M. Pinto, Alexandre da Costa Pereira, Sandhi Maria Barreto, Antonio L. Ribeiro, G Neil Thomas, Gregory Y. H. Lip, Isabela M. Bensenor

**Affiliations:** 1 Universidade de São Paulo Hospital Universitário Centro de Pesquisa Clínica e Epidemiológica São Paulo SP Brasil Hospital Universitário da Universidade de São Paulo, Centro de Pesquisa Clínica e Epidemiológica, São Paulo, SP - Brasil; 2 Universidade Federal de Minas Gerais Faculdade de Medicina Departamento de Clínica Médica Belo Horizonte MG Brasil Faculdade de Medicina da Universidade Federal de Minas Gerais, Departamento de Clínica Médica, Belo Horizonte, MG - Brasil; 3 Universidade Federal de Minas Gerais Faculdade de Medicina Belo Horizonte MG Brasil Faculdade de Medicina da Universidade Federal de Minas Gerais - Programa de Pós-Graduação em Infectologia e Medicina Tropical, Belo Horizonte, MG - Brasil; 4 Faculdade de Medicina da Universidade de São Paulo Hospital das Clínicas Instituto do Coração São Paulo SP Brasil Instituto do Coração do Hospital das Clínicas da Faculdade de Medicina da Universidade de São Paulo - Laboratório de Genética e Cardiologia Molecular, São Paulo, SP - Brasil; 5 Universidade Federal de Minas Gerais Belo Horizonte MG Brasil Universidade Federal de Minas Gerais, Belo Horizonte, MG - Brasil; 6 UFMG Hospital das Clínicas Centro de Telessaúde Belo Horizonte MG Brasil Universidade Federal de Minas Gerais - Centro de Telessaúde - Hospital das Clínicas – UFMG, Belo Horizonte, MG - Brasil; 7 University of Birmingham College of Medical and Dental Sciences Institute of Applied Health Research Birmingham Reino Unido da Grã-Bretanha University of Birmingham College of Medical and Dental Sciences - Institute of Applied Health Research, Birmingham - Reino Unido da Grã-Bretanha; 8 University of Liverpool Liverpool Centre for Cardiovascular Science Liverpool Reino Unido da Grã-Bretanha University of Liverpool - Liverpool Centre for Cardiovascular Science, Liverpool, Merseyside - Reino Unido da Grã-Bretanha; 9 Liverpool Heart & Chest Hospital Liverpool Reino Unido da Grã-Bretanha Liverpool Heart & Chest Hospital, Liverpool - Reino Unido da Grã-Bretanha; 10 Aalborg Universitet Aalborg Thrombosis Research Unit Aalborg Dinamarca Aalborg Universitet - Aalborg Thrombosis Research Unit, Aalborg - Dinamarca

**Keywords:** Fibrilação Atrial, Epidemiologia, Eletrocardiografia/métodos, Anticoagulantes, Estudo Longitudinal, Idoso, Acidente Vascular Cerebral, Embolia

## Abstract

**Fundamento:**

A fibrilação ou flutter atrial (FFA) é a arritmia cardíaca sustentada mais comum. Existem poucos dados sobre a epidemiologia da FFA na América do Sul.

**Objetivo:**

O presente estudo procurou descrever a epidemiologia clínica da FFA e o uso de anticoagulantes na avaliação da linha de base do Estudo Longitudinal da Saúde do Adulto (ELSA-Brasil).

**Métodos:**

Foram analisados dados de 13.260 participantes do ELSA-Brasil. A FFA foi definida pelo eletrocardiograma ou por autorrelato. Modelos de regressão logística foram construídos para analisar fatores associados à FFA. Este estudo também analisou se idade e sexo estavam associados ao uso de anticoagulantes para evitar acidente vascular cerebral. O nível de significância foi de 5%.

**Resultados:**

A idade mediana foi de 51 anos, e 7.213 (54,4%) participantes eram mulheres. A FFA foi detectada em 333 (2,5%) participantes. O aumento da idade (razão de chances [RC]:1,05; intervalo de confiança de 95% [IC95%]: 1,04-1,07), hipertensão (RC:1,44; IC95%:1,14-1,81) coronariopatia (RC: 5,11; IC95%:3,85–6,79), insuficiência cardíaca (RC:7,37; IC95%:5,00–10,87) e febre reumática (RC:3,38; IC95%:2,28–5,02) foram associadas à FFA. Dos 185 participantes com FFA e pontuação no CHA_2_DS_2_-VASc≥2, apenas 20 (10,8%) usavam anticoagulantes (50,0% entre aqueles com FFA no eletrocardiograma de linha de base). O uso de anticoagulantes nesse grupo foi associado a maior idade (1,8% vs 17,7% naqueles com idade ≤ 54 e ≥ 65 anos, respectivamente; p=0,013). Observou-se uma tendência ao menor uso de anticoagulantes em mulheres (7,1% vs. 16,4% em mulheres e homens, respectivamente; p=0,055).

**Conclusões:**

No recrutamento do ELSA-Brasil, 2,5% dos participantes tinham FFA. O baixo uso de anticoagulantes era comum, o que representa um desafio para os cuidados de saúde nesse cenário.

## Introdução

A fibrilação atrial é a arritmia cardíaca sustentada mais comum, com um risco vitalício aproximado de 25%^1^ e um total de 33,3 milhões de casos globalmente.^2^ Em São Paulo, Brasil, um estudo populacional^3^ identificou que 3,2% dos homens e 2,0% das mulheres com idade igual ou superior a 65 anos tinham fibrilação atrial. Outro grande banco de dados de pacientes em atenção primária com idade igual ou superior a 5 anos (71% com idade ≥ 40 anos), em Minas Gerais, no Brasil,^4^ identificou que 2,4% dos homens e 1,3% das mulheres tinham fibrilação atrial. Fatores de risco para fibrilação ou flutter atrial incluem idade, hipertensão, diabetes, tabagismo, obesidade, insuficiência cardíaca, doença valvar, e infarto do miocárdio.^5^^-^^7^

É recomendada a terapia de prevenção de tromboembolismo baseada em risco (incluindo acidente vascular cerebral) para pacientes com fibrilação ou flutter atrial (FFA).^8^^,^^9^ As pontuações CHA_2_DS_2_-VASc^10^ atualmente são a principal estratégia para avaliar o risco de tromboembolismo nesses indivíduos.^11^ A terapia antitrombótica oral é recomendada para pacientes com uma pontuação CHA_2_DS_2_-VASc ≥ 2.^9^

A terapia anticoagulante oral, entretanto, ainda representa um desafio para a prática clínica,^12^^,^^13^ e uma proporção significativa da carga de morbidade da FFA ainda está associada ao acidente vascular cerebral incidente,^14^ especialmente em mulheres.^15^^,^^16^ Isso é ainda mais importante no Brasil, onde, embora os índices de incidentes de acidente vascular cerebral padronizada para a idade do país tenham caídos nos últimos anos,^17^ a mortalidade por acidente vascular cerebral é ainda alta, comparada a outros países sul-americanos, e tem um impacto maior nos estados menos desenvolvidos do país.^17^^,^^18^

O estudo Longitudinal de Saúde do Adulto (ELSA-Brasil) é uma grande coorte multicêntrica de indivíduos entre 35 e 74 anos de seis cidades brasileiras, com o objetivo de estudar a incidência e os fatores associados às doenças cardiovasculares e ao diabetes. O objetivo do presente estudo é descrever a frequência da FFA na linha de base do ELSA-Brasil e o uso de medicamentos de prevenção de acidente vascular cerebral nos participantes com FFA e com uma pontuação CHA_2_DS_2_-VASc ≥ 2.

## Métodos

### Configuração e desenho do estudo

O desenho do ELSA-Brasil^19^ e o perfil de coorte^20^ estão detalhados em outro local. Resumidamente, o ELSA-Brasil é um estudo coorte de 15.105 funcionários públicos de seis cidades brasileiras (São Paulo, Belo Horizonte, Porto Alegre, Salvador, Rio de Janeiro, e Vitória). As avaliações de linha de base foram realizadas entre agosto de 2008 e dezembro de 2010 e incluíram entrevistas presenciais realizadas por pessoal treinado, utilizando exames clínicos, laboratoriais e de imagem. Desde a linha de base, todos os participantes receberam um contato telefone anual para acompanhamento. Quatro anos após o recrutamento (2012-2014), todos os participantes foram convidados a passar por uma reavaliação local, que incluiu questionários e avaliações clínicas e laboratoriais. Essa reavaliação teve a presença de 14.014 (92,8%) participantes. O termo de consentimento informado foi obtido de todos os participantes. O protocolo do estudo está de acordo com a Resolução Nº 466/2012 do Conselho Nacional de Saúde e foi aprovado pelo Comitê de Análise Institucional de cada centro participante.

### Diagnóstico da fibrilação ou flutter atrial

Os métodos de registro dos traçados de eletrocardiogramas (ECG) no ELSA-Brasil estão detalhados em outro artigo.^21^ O eletrocardiograma na linha de base foi realizado em cada centro, utilizando-se um dispositivo Burdick Atria 6100, calibrado a 10 mm/mV e uma velocidade de 25 mm/segundo. Os registros foram transmitidos ao centro de leitura no Centro de Investigação em Minas Gerais. As análises seguiram o sistema de Glasgow,^22^ e foram codificadas de acordo com o Minnesota Coding System. Códigos selecionados (incluindo FFA) foram analisados manualmente por uma equipe treinada. Além disso, durante a reavaliação local realizada em 2012-2014, a seguinte pergunta foi feita aos participantes: *“Algum médico já disse que você tem/tinha fibrilação atrial?”* Os participantes que responderam “sim” a essa pergunta também tiveram que responder *“Quantos anos você tinha a primeira vez que um médico disse que você tem/tinha fibrilação atrial?”*

O presente estudo definiu o diagnóstico de FFA na linha de base se o participante (a) tivesse um registro de eletrocardiograma com FFA na avaliação de linha de base do ELSA-Brasil (n=48) ou (b) indicasse que tinha tido um diagnóstico de fibrilação atrial numa idade inferior à sua, no momento do recrutamento para o ELSA-Brasil (n=285). A maioria das análises são apresentadas para todos os casos de FFA e de acordo com cada critério de definição.

### Amostra do estudo

Este estudo incluiu participantes do ELSA-Brasil para os quais o diagnóstico de FFA na linha de base poderia ser avaliado. Dos 15.105 participantes do ELSA-Brasil, 663 (4,4%) foram excluídos por não terem uma leitura válida de eletrocardiograma na linha de base, nem uma resposta válida sobre FFA durante a reavaliação local de 2012-2014, e 1.182 (7,8%) foram excluídos porque tinham uma leitura válida de eletrocardiograma na linha de base sem FFA, mas não tinham uma resposta válida sobre FFA na reavaliação local de 2012-2014 (prejudicando a avaliação de FFA paroxística). Portanto, nossa amostra principal consistiu em 13.260 (87,8%) participantes.

### Outras variáveis

Idade, sexo, raça, grau de escolaridade, renda familiar mensal, e tabagismo foram autorrelatados e estratificados adequadamente. A renda mensal foi convertida em dólares americanos (utilizando uma taxa de câmbio de BRL 2,00=USD 1,00, com base na taxa de câmbio na linha de base). A raça foi definida, conforme utilizada no Censo Nacional Brasileiro, ou seja, preta, parda, branca, amarela ou indígena. As medições antropométricas no ELSA-Brasil foram avaliadas utilizando técnicas padronizadas,^23^ e o índice de massa corporal (IMC) foi calculado dividindo-se o pelo pela altura ao quadrado. O IMC foi categorizado como normal (< 25 kg/m^2^), sobrepeso (≥ 25 kg/m^2^ e < 30 kg/m^2^), e obesidade (≥ 30 kg/m^2^). Hipertensão foi definida pelo relato de uso de medicamento anti-hipertensivos, pressão arterial sistólica ≥ 140 mmHg, ou pressão arterial diastólica ≥ 90 mmHg no momento da avaliação de linha de base do ELSA-Brasil. Diabetes foi definido pelo histórico médico de diabetes mellitus, relato de uso de medicamentos para tratar diabetes mellitus, glicemia em jejum ≥ 126 mg/dl, níveis de HbA1c ≥ 6,5%, ou um teste oral de tolerância à glicose de 2 horas ≥ 200 mg/dl. Dislipidemia foi definida pelo relato do uso de tratamento de redução lipídica ou um nível de colesterol LDL ≥ 130 mg/dl. Obesidade abdominal foi definida como uma circunferência > 88 cm em mulheres e 102 cm em homens. Diagnósticos médicos anteriores de insuficiência cardíaca, acidente vascular cerebral, febre reumática, e evento tromboembólico foram avaliados a partir de autorrelato. Coronariopatia foi definida por um diagnóstico médico anterior de infarto do miocárdio ou angina, ou por revascularização coronária anterior. As aferições de pressão tornozelo-braquial para cálculo de índice tornozelo-braquial (ITB) são descritas detalhadamente por Miname et al.^24^. De acordo com os achados desse estudo, o ITB foi calculado como a proporção entre a pressão sistólica mínima do tornozelo e a pressão sistólica máxima no braço, para obter uma sensibilidade maior. Doença arterial periférica foi definida quando o ITB era < 1,0.^24^^,^^25^ Para dados sobre o uso de medicamentos, além dos questionários, solicitou-se que os pacientes trouxessem para a linha de base, todos os medicamentos, com receita médica ou não, que eles estivessem tomando no momento.^26^ O uso de anticoagulantes foi definido como o uso de um medicamento com código *Anatomical Therapeutic Chemical* (ATC) B01AA ou B01AB. O uso de antiagregantes plaquetários foi definido como o uso de medicamentos com código ATC B01AC. Para participantes sem coronariopatia ou acidente vascular cerebral, o risco de doença cardiovascular aterosclerótica (DCVAS) de 10 anos foi calculado de acordo com as diretrizes de Avaliação de Risco Cardiovascular da ACC/AHA de 2013.^27^ As pontuações CHA_2_DS_2_-VASc foram calculadas de acordo com Lip et al.^11^ e categorizadas como 0 ou 1 pontos, 2 ou 3 pontos, e ≥ 4 pontos.

### Análise estatística

As variáveis categóricas são apresentadas como contagens e proporções, e são comparadas usando-se o teste Qui-quadrado ou o teste exato de Fisher. As variáveis contínuas (idade, índice de massa corporal, índice tornozelo-braquial, e risco de DCVAS de 10 anos) têm distribuição não normal (p<0,001 para todas, usando o teste Anderson-Darling), e são apresentadas como medianas e faixas interquartis, e são comparadas entre os grupos, usando o teste U de Mann-Whitney. Modelos de regressão logística brutos padronizados para a idade e o sexo foram construídos para analisar se o diagnóstico de FFA na linha de base do ELSA-Brasil estava associado a idade, sexo, raça, grau de escolaridade, renda mensal, tabagismo, diagnósticos de hipertensão, diabetes e dislipidemia, índice de massa corporal, obesidade abdominal, doença arterial periférica, coronariopatia, insuficiência cardíaca, acidente vascular cerebral, febre reumática, e um risco de DCC de 10 anos > 10%. A regressão logística binária foi usada para analisar a associação com todos os casos de FFA, enquanto a regressão logística multinomial foi usada para analisar a associação com casos de FFA de acordo com cada critério de definição (por registro de eletrocardiograma ou autorrelato) separadamente. O nível de significância foi definido em 5%, e o software R, versão 3.5.1, foi usado em todas as análises.

## Resultados

Entre os 13.260 participantes incluídos nas análises, 333 (2,5%) tinham FFA na linha de base do ELSA-Brasil, 176/7.213 (2,4%) eram mulheres e 157/6.047 (2,6%) eram homens. De acordo com a faixa etária, a frequência de diagnóstico de FFA foi 1,2%, 2,2%, 2,9%, e 5,4% para as idades < 45, 45-54, 55-64, e > 64 anos, respectivamente. A Tabela 1 mostra as características das amostras do estudo de acordo com a presença de diagnóstico de FFA na linha de base do ELSA-Brasil. Os indivíduos não brancos representaram 47,9% da amostra, e a maioria dos participantes tinham ensino superior completo ou mais.

**Tabela 1:** Características das amostras do estudo de acordo com a presença de diagnóstico de FFA na linha de base do ELSA-Brasil

		Sem fibrilação ou flutter atrial. (N=12.927)	Fibrilação ou flutter atrial				
			Por registro de eletrocardiograma (N=48)	Apenas por autorrelato (N=285)	Todos os casos de FFA (N=333)	Total (N=13.260)	p-valor
Idade (anos; mediana [P25 - P75])		51,0 [45,0 – 58,0]	61,5 [56,0 – 71,0]	54,0 [49,0 – 62,0]	56,0 [49,0 – 63,0]	51,0 [45,0 – 58,0]	<0,001 ‡
Sexo feminino (N (%))		7.037 (54,4%)	18 (37,5%)	158 (55,4%)	176 (52,9%)	7.213 (54,4%)	0,61 †
**Raça (N (%))**							
	Branco	6.652 (52,1%)	29 (61,7%)	154 (54,6%)	183 (55,6%)	6.835 (52,1%)	0,039 †
	Pardo	3.608 (28,2%)	11 (23,4%)	74 (26,2%)	85 (25,8%)	3.693 (28,2%)	
	Negro	2.069 (16,2%)	7 (14,9%)	35 (12,4%)	42 (12,8%)	2.111 (16,1%)	
	Outros	449 (3,5%)	0 (0,0%)	19 (6,7%)	19 (5,8%)	468 (3,6%)	
**Grau de escolaridade (N (%))**							
	Ensino médio incompleto	1.529 (11,8%)	13 (27,1%)	34 (11,9%)	47 (14,1%)	1.576 (11,9%)	0,10 †
	Ensino médio completo	4.443 (34,4%)	12 (25,0%)	85 (29,8%)	97 (29,1%)	4.540 (34,2%)	
	Ensino superior ou mais	6.955 (53,8%)	23 (47,9%)	166 (58,2%)	189 (56,8%)	7.144 (53,9%)	
**Renda mensal (N (%))**							
	< USD$ 1245	3.357 (26,1%)	15 (31,2%)	58 (20,4%)	73 (22,0%)	3.430 (26,0%)	0,001 †
	USD$ 1245-3319	5.679 (44,1%)	12 (25,0%)	115 (40,5%)	127 (38,3%)	5.806 (43,9%)	
	≥ USD$ 3320	3.845 (29,9%)	21 (43,8%)	111 (39,1%)	132 (39,8%)	3.977 (30,1%)	
**Tabagismo (N (%))**							
	Nunca fumou	7.467 (57,8%)	29 (60,4%)	161 (56,5%)	190 (57,1%)	7.657 (57,7%)	0,75 †
	Ex-fumante	3.822 (29,6%)	13 (27,1%)	91 (31,9%)	104 (31,2%)	3.926 (29,6%)	
	Fumante	1.637 (12,7%)	6 (12,5%)	33 (11,6%)	39 (11,7%)	1.676 (12,6%)	
Hipertensão (N (%))		4.463 (34,5%)	30 (62,5%)	136 (47,7%)	166 (49,8%)	4.629 (34,9%)	<0,001 †
Diabetes (N (%))		2.438 (18,9%)	15 (31,2%)	66 (23,2%)	81 (24,3%)	2.519 (19,0%)	0,015 †
Dislipidemia (N (%))		7.489 (58,0%)	27 (56,2%)	188 (66,0%)	215 (64,6%)	7.704 (58,1%)	0,019 †
Índice de massa corporal (kg/m^2^; mediana [P25 - P75])		26,3 [23,7 – 29,5]	27,4 [25,4 – 30,4]	26,2 [23,9 – 30,2]	26,5 [24,1 – 30,3]	26,3 [23,7 – 29,6]	0,35 ‡
**Classificação do índice de massa corporal (N (%))**							
	Normal	4.791 (37,1%)	10 (20,8%)	104 (36,5%)	114 (34,2%)	4.905 (37,0%)	0,057 †
	Sobrepeso	5.238 (40,5%)	24 (50,0%)	102 (35,8%)	126 (37,8%)	5.364 (40,5%)	
	Obesidade	2.892 (22,4%)	14 (29,2%)	79 (27,7%)	93 (27,9%)	2.985 (22,5%)	
Obesidade abdominal (N (%))		4.656 (36,0%)	20 (41,7%)	113 (39,6%)	133 (39,9%)	4.789 (36,1%)	0,16 †
Índice tornozelo-braquial (mediana [P25 - P75])		1,18 [1,12 – 1,23]	1,13 [1,04 – 1,20]	1,17 [1,11 – 1,24]	1,16 [1,10 – 1,23]	1,18 [1,12 – 1,23]	0,008 ‡
Doença arterial periférica (N (%))		403 (3,1%)	6 (12,5%)	12 (4,2%)	18 (5,4%)	421 (3,2%)	0,025 †
Uso de anticoagulantes (N (%))		37 (0,3%)	19 (39,6%)	5 (1,8%)	24 (7,2%)	61 (0,5%)	<0,001 †
Uso de antiagregantes plaquetários (N (%))		706 (5,5%)	7 (14,6%)	45 (15,8%)	52 (15,6%)	758 (5,7%)	<0,001 †
Uso de anticoagulantes e/ou antiagregantes plaquetários (N (%))		741 (5,7%)	26 (54,2%)	50 (17,5%)	76 (22,8%)	817 (6,2%)	<0,001 †
Coronariopatia (N (%))		536 (4,2%)	7 (14,6%)	67 (23,5%)	74 (22,2%)	610 (4,6%)	<0,001 †
Insuficiência cardíaca (N (%))		160 (1,2%)	10 (20,8%)	26 (9,2%)	36 (10,8%)	196 (1,5%)	<0,001 †
Acidente vascular cerebral (N (%))		153 (1,2%)	1 (2,1%)	7 (2,5%)	8 (2,4%)	161 (1,2%)	0,067 †
Febre reumática (N (%))		352 (2,7%)	7 (14,6%)	23 (8,1%)	30 (9,0%)	382 (2,9%)	<0,001 †
Evento tromboembólico (N (%))		198 (1,5%)	4 (8,3%)	5 (1,8%)	9 (2,7%)	207 (1,6%)	0,11 †
Risco de DCVAS de 10 anos (mediana [P25 - P75])		2,8% [1,1% −7,1%]	11,8% [4,2% – 18,5%]	2,7% [1,4% – 7,9%]	3,4% [1,5% - 10,4%]	2,8% [1,1% – 7,2%]	0,001 ‡
Risco de DCVAS de 10 anos > 10% (N (%))		2075 (16,9%)	23 (57,5%)	41 (19,2%)	64 (25,2%)	2139 (17,1%)	0,001 †

O risco de DCVAS de 10 anos é definido apenas para participantes sem histórico de coronariopatia ou acidente vascular cerebral. FFA: Fibrilação ou flutter atrial. DCVAS: Doença cardiovascular aterosclerótica. Os p-valores são apresentados para comparação entre o grupo sem fibrilação ou flutter atrial (N=12.927) e todos os casos de FFA (N=333), utilizando os testes

† Qui-quadrado ou

‡ U de Mann-Whitney.

A Tabela 2 mostra razões de chance padronizadas para idade e sexo para a associação com a presença de diagnóstico de FFA na linha de base do ELSA-Brasil. Considerando todos os casos de FFA, idade mais alta (p<0,001), renda mais alta (p=0,044), e diagnósticos de hipertensão (p=0,002), coronariopatia (p<0,001), insuficiência cardíaca (p<0,001), e febre reumática (p<0,001) foram positivamente associados a FFA nos modelos padronizados. Restringindo os casos aos 48 participantes que apresentaram FFA no eletrocardiograma de linha de base, idade mais alta (p<0,001), sexo masculino (p=0,037), e diagnósticos de doença arterial periférica (p=0,026), insuficiência cardíaca (p<0,001), e febre reumática (p<0,001) foram positivamente associados a FFA nos modelos padronizados. A Tabela suplementar 1 mostra os resultados de modelos brutos para essas associações.

**Tabela 2:** Razões de chance padronizadas para idade e sexo (intervalos de confiança de 95%) para a associação com fibrilação ou flutter atrial na linha de base do ELSA-Brasil

			Fibrilação ou flutter atrial	
Variável		Por registro de eletrocardiograma (N=48)	Apenas por autorrelato (N=285)	Todos os casos de FFA (N=333)
Idade (incrementos de um ano)		1,12 [1,09 – 1,16]	1,04 (1,03 – 1,06) †	1,05 (1,04 – 1,07) †
Sexo feminino		0,53 (0,30 – 0,96) †	1,05 (0,83 – 1,33)	0,95 (0,76 – 1,18)
**Raça** *				
	Branco	1,0 (Referência)	1,0 (Referência)	1,0 (Referência)
	Pardo	0,92 (0,45 – 1,85)	0,95 (0,72 – 1,26)	0,94 (0,72 – 1,22)
	Negro	(0,43 – 2,27)	0,76 (0,52 – 1,10)	0,79 (0,56 – 1,10)
**Grau de escolaridade**				
	Ensino médio incompleto	1,73 (0,87 – 3,43)	0,79 (0,54 – 1,15)	0,92 (0,66 – 1,27)
	Ensino médio completo	1,12 (0,55 – 2,28)	0,86 (0,66 – 1,12)	0,88 (0,69 – 1,13)
	Ensino superior ou mais	1,0 (Referência)	1,0 (Referência)	1,0 (Referência)
**Renda mensal**				
	< USD$ 1245	1,27 (0,64 – 2,51)	0,67 (0,48 – 0,93) †	0,74 (0,55 – 0,99) †
	USD$ 1245-3319	0,61 (0,29 – 1,25)	0,80 (0,61 – 1,04)	0,77 (0,60 – 0,99) †
	≥ USD$ 3320 (N (%))	1,0 (Referência)	1,0 (Referência)	1,0 (Referência)
**Tabagismo**				
	Nunca fumou	(Referência)	(Referência)	1,0 (Referência)
	Ex-fumante	0,57 (0,29 - 1,12)	0,98 (0,75 – 1,27)	0,90 (0,70 – 1,16)
	Fumante	1,00 (0,42 – 2,40)	0,93 (0,64 – 1,36)	0,93 (0,65 – 1,32)
Hipertensão		1,65 (0,89 – 3,03)	1,41 (1,10 – 1,81) †	1,44 (1,14 – 1,81) †
Diabetes		1,11 (0,60 – 2,08)	1,06 (0,80 – 1,41)	1,07 (0,82 – 1,39)
Dislipidemia		0,66 (0,37 – 1,18)	1,22 (0,95 – 1,57)	1,10 (0,87 – 1,39)
**Classificação do índice de massa corporal**				
	Normal	1,0 (Referência)	1,0 (Referência)	1,0 (Referência)
	Sobrepeso	1,88 (0,90 – 3,95)	0,85 (0,64 – 1,12)	0,94 (0,72 – 1,21)
	Obesidade	2,21 (0,97 – 5,00)	1,19 (0,88 – 1,60)	1,27 (0,96 – 1,68)
Obesidade abdominal		1,15 (0,63 – 2,08)	1,04 (0,81 – 1,33)	1,05 (0,83 – 1,32)
Doença arterial periférica		2,72 (1,13 – 6,54) †	1,16 (0,64 – 2,10)	1,44 (0,88 – 2,35)
Doença arterial coronariana		1,87 (0,81 – 4,28)	5,99 (4,44 – 8,09) †	5,11 (3,85 – 6,79) †
Insuficiência cardíaca		11,67 (5,58 – 24,40) †	6,51 (4,19 – 10,11) †	7,37 (5,00 – 10,87) †
Acidente vascular		0,90 (0,12 – 6,67)	1,65 (0,76 – 3,57)	1,51 (0,73 – 3,13)
Febre reumática		5,75 (2,55 – 12,98) †	3,02 (1,94 – 4,70) †	3,38 (2,28 – 5,02) †
Risco de DCVAS de 10 anos > 10%		1,56 (0,63 – 3,85)	0,78 (0,50 – 1,21)	0,95 (0,65 – 1,40)

* A proporção pequena de indivíduos de outras raças na amostra impossibilitou a estimativa. FFA: Fibrilação ou flutter atrial. DCVAS: Doença cardiovascular aterosclerótica. As razões de chance e os p-valores foram obtidos a partir de modelos de regressão logística padronizados para idade e sexo.

† p<0,05.

Foi possível calcular as pontuações CHA_2_DS_2_-VASc para 331 (99,4%) dos 333 participantes com diagnóstico de FFA na linha de base (Tabela 3). Como esperado, os indivíduos com pontuações CHA_2_DS_2_-VASc mais altas eram mais velhos (p<0,001), tinham uma probabilidade mais alta de serem mulheres (p=0,002), e apresentaram um risco cardiovascular mais alto (p<0,001). Entre os 185 participantes com FFA e pontuação no CHA_2_DS_2_-VASc ≥2, apenas 20 (10,8%) faziam tratamento com anticoagulantes (16/32; 50,0% quando os casos eram restritos aos 48 participantes que apresentaram FFA no eletrocardiograma de linha de base).

**Tabela 3:** Características dos participantes com fibrilação ou flutter atrial na linha de base do ELSA-Brasil de acordo com as pontuações CHA2DS2-VASc

	Fibrilação ou flutter atrial						
	Por registro de eletrocardiograma		Apenas por autorrelato		Todos os casos de FFA		
	Pontuação CHA2DS2-VASc < 2 (N=16)	Pontuação CHA2DS2-VASc ≥ 2 (N=32)	Pontuação CHA2DS2-VASc < 2 (N=130)	Pontuação CHA2DS2-VASc ≥ 2 (N=153)	Pontuação CHA2DS2-VASc < 2 (N=146)	Pontuação CHA2DS2-VASc ≥ 2 (N=185)	p-valor
Idade (anos; mediana [P25 - P75])	56,0 [49,5 – 61,2]	67,5 [58,0 – 71,2]	49,5 [45,0 – 56,0]	59,0 [53,0 – 66,0]	50,0 [45,0 – 57,0]	60,0 [53,0 – 68,0]	<0,001 ‡
Sexo feminino (N (%))	3 (18,8%)	15 (46,9%)	60 (46,2%)	97 (63,4%)	63 (43,2%)	112 (60,5%)	0,002 †
Coronariopatia (N (%))	0 (0,0%)	7 (21,9%)	2 (1,5%)	64 (41,8%)	2 (1,4%)	71 (38,4%)	<0,001 †
Acidente vascular cerebral (N (%))	0 (0,0%)	1 (3,1%)	0 (0,0%)	7 (4,6%)	0 (0,0%)	8 (4,3%)	0,010 ¥
Risco de DCVAS em 10 anos > 10% (N (%))	7 (43,8%)	16 (66,7%)	15 (11,7%)	26 (30,6%)	22 (15,3%)	42 (38,5%)	<0,001 †
DCVAS anterior ou Risco de DCVAS em 10 anos anterior > 10%	7 (43,8%)	24 (75,0%)	17 (13,1%)	94 (61,4%)	24 (16,4%)	118 (63,8%)	<0,001 †
Uso de anticoagulantes (N (%))	3 (18,8%)	16 (50,0%)	1 (0,8%)	4 (2,6%)	4 (2,7%)	20 (10,8%)	0,005 ¥
Uso de antiagregantes plaquetários (N (%))	2 (12,5%)	5 (15,6%)	2 (1,5%)	43 (28,1%)	4 (2,7%)	48 (25,9%)	<0,001 †

O risco de DCVAS de 10 anos é definido apenas para participantes sem histórico de coronariopatia ou acidente vascular cerebral. FFA: Fibrilação ou flutter atrial. DCVAS: Doença cardiovascular aterosclerótica. Os p-valores são apresentados para comparação entre os grupos com pontuação CHA_2_DS_2_-VASc < 2 (N=146) e com pontuação CHA_2_DS_2_-VASc ≥ 2 (N=185) entre todos os casos de FFA, utilizando os testes

† Qui-quadrado,

‡ U de Mann-Whitney U ou

¥ exato de Fisher.

A Tabela 4 mostra a prevalência do uso de anticoagulantes orais em participantes com FFA e uma pontuação CHA_2_DS_2_-VASc ≥ 2, de acordo com idade e sexo. Considerando todos os casos de FFA, o uso de anticoagulantes foi mais frequente nos participantes com idade mais alta (p=0,013), e uma tendência para uso mais baixo de anticoagulantes em mulheres (p=0,055). A Tabela suplementar 2 mostra que a frequência do uso de anticoagulantes em homens era numericamente mais alta do que em mulheres para todas as faixas etárias. Entretanto, não foram descobertas diferenças significativas nem ao analisar todos os casos de FFA nem ao restringi-los aos participantes que apresentaram FFA no eletrocardiograma da linha de base.

**Tabela 4:** Frequência do uso de anticoagulantes, de acordo com idade e sexo, em participantes com fibrilação ou flutter atrial e uma pontuação CHA_2_DS_2_-VASc ≥ 2, na linha de base do ELSA-Brasil

			Fibrilação ou flutter atrial e uma pontuação CHA_2_DS_2_-VASc ≥ 2		
Variável		Por registro de eletrocardiograma (N=32)	Apenas por autorrelato (N=153)	Todos os casos de FFA (N=185)	p-valor
**Idade (anos)**					0,013
	≤ 54	1 / 3 (33,3%)	0 / 52 (0,0%)	1 / 55 (1,8%)	
	55 – 64	6 / 11 (54,5%)	2 / 57 (3,5%)	8 / 68 (11,8%)	
	≥ 65	9 / 18 (50,0%)	2 / 44 (4,5%)	11 / 62 (17,7%)	
**Sexo**					0,055
	Masculino	9 / 17 (52,9%)	3 / 56 (5,4%)	12 / 73 (16,4%)	
	Feminino	7 / 15 (46,7%)	1 / 97 (1,0%)	8 / 112 (7,1%)	

A frequência do uso de anticoagulantes, de acordo com idade e sexo, considerando todos os casos de FFA com uma pontuação de CHA_2_DS_2_-VASc ≥ 2 foi comparada usando o teste exato de Fisher.

## Discussão

### Contextualização e discussão dos principais achados

Em nossa amostra de coorte, 2,5% dos participantes incluídos apresentaram FFA na linha de base. Idade, sexo masculino, renda, e diagnósticos de hipertensão, coronariopatia, doença arterial periférica, insuficiência cardíaca e febre reumática foram associados positivamente ao FFA. Segundo, a maioria dos participantes com FFA e uma pontuação CHA_2_DS_2_-VASc ≥ 2 não foi tratada com anticoagulantes e/ou antiagregantes plaquetários. Em terceiro lugar, o uso de anticoagulante oral foi mais comum naqueles com idade mais alta, enquanto as mulheres eram tratadas menos frequentemente.

A comparação da prevalência de FFA em amostras diferentes é desafiadora devido às heterogeneidades no desenho do estudo, no cenário do estudo, e na distribuição de idade e sexo no recrutamento. Uma análise da epidemiologia global da fibrilação atrial^28^ detectou uma prevalência mundial relatada variando entre 0,1% e 6,0%, dependendo da faixa etária e do sexo analisados. Embora este estudo relate uma frequência de diagnóstico de FFA semelhante na amostra, quando comparado com os estudos brasileiros citados,^3^^,^^4^ esses estudos têm diferenças marcadas e são complementares. Por exemplo, Kawabata-Yoshihara et al.^3^ realizaram um estudo de recrutamento porta a porta sistemático de indivíduos com idade igual ou superior a 65 anos, enquanto Marcolino et al.^4^ usou um grande banco de dados de eletrocardiogramas da Rede de Teleassistência de Minas Gerais, composto de indivíduos com idade igual ou superior a 5 anos que receberam cuidados médicos em unidades de atenção primária de saúde. Ambos os estudos consideraram o diagnóstico de FFA de acordo com os registros de eletrocardiograma na avaliação. Ao comparar os achados desses estudos com os achados do presente estudo, detectou-se uma frequência mais alta de FFA quando faixas etárias semelhantes foram comparadas. Provavelmente isso foi influenciado pelas definições de caso, já que o presente estudo também incluiu um diagnóstico médico autorrelatado como critério diagnóstico. Entretanto, considerar apenas os traçados de eletrocardiograma durante a avaliação e excluir o histórico médico das definições pode levar a uma especificidade mais alta e reduzir erros de informação autorrelatada, mas também pode subestimar os índices de prevalência devido ao sub-reconhecimento da fibrilação atrial paroxística.

A associação entre insuficiência cardíaca e fibrilação atrial tem importância clínica. Healey et al.^12^ analisaram dados de 15.400 indivíduos com FFA em 47 países. Depois de um ano de acompanhamento, ocorreram 11% de mortes no coorte, e a insuficiência cardíaca foi a causa de morte mais comum (30%). Da mesma maneira, os indivíduos com doença arterial periférica e FFA têm um risco mais alto de acidente vascular cerebral, comparados com aqueles que só têm FFA.^29^ É importante observar que, em décadas recentes, a doença cardíaca reumática tem diminuído como causa de mortalidade no Brasil,^30^ e, embora novos casos no país sejam menos comuns, pessoas atualmente de meia idade e adultos mais velhos podem ter tido febre reumática durante a infância. Portanto, a prevalência e a mortalidade devidas à doença cardíaca reumática no Brasil não podem ser desprezadas,^31^ o que é reforçado por nosso achado de que 9,0% dos indivíduos com FFA nessa amostra de estudo (e 2,9% em geral) apresentaram um histórico médico de febre reumática.

Um achado notável do presente estudo foi que 89,2% de todos os participantes com FFA e uma pontuação CHA_2_DS_2_-VASc ≥ 2 não foram tratados com anticoagulante. Isso é mais importante, considerando que os participantes do ELSA-Brasil tinham um grau de escolaridade médio e renda mais altos, quando comparados à população brasileira geral.^20^ Embora os diagnósticos autorrelatados possam ter influenciado nossos achados de baixos índices de prevenção de acidente vascular cerebral, quando limitamos as análises a participantes com FFA documentado no registro de eletrocardiograma de linha de base, metade deles ainda não tinha recebido anticoagulante.

Embora preocupantes, esses baixos índices de prescrição para prevenção de acidente vascular cerebral não são exclusividade de nossa amostra. Um estudo semelhante realizado por Healey et al.^12^ descreveu que, por região geográfica, de 32% (América do Norte, Europa Ocidental e Oriente Médio) a 70% (China) pacientes com indicação por diretriz não receberam tratamento com anticoagulante oral. Na América do Sul, onde o ELSA-Brasil está localizado, a frequência da não prescrição era 55%. Ogilvie et al.^32^ relataram uma análise sistemática de estudos relatando o uso de anticoagulantes orais em casos de fibrilação atrial fora de ensaios clínicos. Eles identificaram que 21/29 (72,4%) estudos em pacientes com acidente vascular cerebral ou ataque isquêmico transitório prévio, bem como 5/9 (55,6%) estudos em pacientes com alto risco tromboembólico, de acordo com escores de risco, relataram níveis de prescrição inferiores a 60%.

A subprescrição de medicamentos para a prevenção de acidente vascular cerebral parece ser um problema mais grave em mulheres,^33^ o que está de acordo com nossos dados. Khurshid et al.^34^ analisaram dados médicos eletrônicos de 4.388 pacientes com fibrilação atrial e detectaram que, comparadas aos homens, as mulheres receberam menos tratamento com anticoagulantes 1, 3 e 6 meses após o diagnóstico de fibrilação atrial. Além disso, Emdin et al.^35^ realizaram uma análise sistemática e metanálise de 30 estudos coorte e identificaram que, comparadas aos homens, mulheres com fibrilação atrial tinham um risco mais alto de mortalidade global, mortalidade cardiovascular, e acidente vascular cerebral. Esses autores destacam que, além da subutilização de medicamentos para prevenção de acidente vascular cerebral, outras explicações para resultados piores, tais como os índices mais altos de efeitos adversos dos tratamentos com anticoagulantes^36^ e antiarrítmicos^37^ em mulheres são igualmente plausíveis. Além disso, alguns autores destacaram um controle anticoagulante pior em mulheres que utilizam a varfarina. Por exemplo, Sullivan et al.^38^ analisaram dados de 4.060 participantes do estudo *Atrial Fibrillation Follow-up Investigation of Rhythm Management* (AFFIRM - Investigação de monitoramento da fibrilação atrial para controle do ritmo cardíaco), e concluíram que, comparadas aos homens, as mulheres em tratamento com varfarina passaram consideravelmente mais tempo utilizando doses inferiores à faixa terapêutica da razão normalizada internacional (RNI) (29% vs 26%). Também é importante notar que, a avaliação da linha de base do ELSA-Brasil ocorreu de 2008 a 2010. Nesse momento, o uso de anticoagulantes orais de ação direta para a prevenção de acidente vascular cerebral nos casos de FFA era muito raro.

### Pontos fortes e limitações

Este estudo realmente tem alguns pontos fortes. Analisamos dados de uma amostra multicêntrica grande de indivíduos que não foram recrutados de cenários clínicos. Isso reduz possíveis vieses no estudo de fatores associados à fibrilação atrial e diagnósticos, e a prescrição de medicamentos para a prevenção de acidente vascular cerebral, aproximando nossa validade externa à população geral. O protocolo abrangente do ELSA-Brasil permitiu a análise do uso de medicamento de acordo com as pontuações de risco tromboembólico CHA_2_DS_2_-VASc. O estudo também deve ser interpretado dentro de seu contexto. Conforme afirmado anteriormente, esta definição do estudo de caso pode ter uma tendência a classificações erradas por informações autorrelatadas. Embora essa estratégia minimize o sub-reconhecimento da fibrilação atrial paroxística, é importante admitir que casos autorrelatados representaram uma proporção importante de todos os casos de FFA, e isso pode ter influenciado alguns de nossos resultados. Os dados sobre a frequência de indivíduos dentro da faixa terapêutica de RNI ou escores de risco de hemorragia não estavam disponíveis. Entretanto, acreditamos que o risco de hemorragia não é suficiente para explicar uma parte significativa da falta de prevenção de acidente vascular cerebral nessa amostra.

## Conclusões

Concluindo, foi identificada uma frequência de diagnóstico de FFA de 2,5% na avaliação de linha de base do ELSA-Brasil. Idade, sexo masculino, renda, e diagnósticos de doença arterial periférica, insuficiência cardíaca e febre reumática foram associados à FFA. Aproximadamente 90% da subamostra dos participantes com FFA e uma pontuação CHA_2_DS_2_-VASc ≥ 2 (50% considerando apenas os diagnosticados por registros de eletrocardiograma) não foram tratados com anticoagulantes.
