# Supplementary material for: Diagnóstico de Fibrilação Atrial na Comunidade Utilizando Eletrocardiograma e Autorrelato: Análise Transversal do ELSA-Brasil
Source: Arq Bras Cardiol. 2021 Sep 1;117(3):426–34. [Article in Portuguese] doi: 10.36660/abc.20190873 (PMC8462958; doi:10.36660/abc.20190873)
Supplement: Translation

# Translation: Atrial Fibrillation Diagnosis using ECG Records and Self-Report in the Community: Cross-Sectional Analysis from ELSA-Brasil

**Authors:** Itamar S. Santos, Paulo A. Lotufo, Luisa Brant, Marcelo M. Pinto, Alexandre da Costa Pereira, Sandhi Maria Barreto, Antonio L. Ribeiro, G Neil Thomas, Gregory Y. H. Lip, Isabela M. Bensenor

**Affiliations:** 1 São Paulo SP Brazil Hospital Universitário da Universidade de São Paulo, Centro de Pesquisa Clínica e Epidemiológica, São Paulo, SP - Brazil; 2 Belo Horizonte MG Brazil Faculdade de Medicina da Universidade Federal de Minas Gerais, Departamento de Clínica Médica, Belo Horizonte, MG - Brazil; 3 Belo Horizonte MG Brazil Faculdade de Medicina da Universidade Federal de Minas Gerais - Programa de Pós-Graduação em Infectologia e Medicina Tropical, Belo Horizonte, MG - Brazil; 4 São Paulo SP Brazil Instituto do Coração do Hospital das Clínicas da Faculdade de Medicina da Universidade de São Paulo - Laboratório de Genética e Cardiologia Molecular, São Paulo, SP - Brazil; 5 Belo Horizonte MG Brazil Universidade Federal de Minas Gerais, Belo Horizonte, MG - Brazil; 6 Belo Horizonte MG Brazil Universidade Federal de Minas Gerais - Centro de Telessaúde - Hospital das Clínicas – UFMG, Belo Horizonte, MG - Brazil; 7 Birmingham Reino Unido da Grã-Bretanha University of Birmingham College of Medical and Dental Sciences - Institute of Applied Health Research, Birmingham - Reino Unido da Grã-Bretanha; 8 Liverpool Reino Unido da Grã-Bretanha University of Liverpool - Liverpool Centre for Cardiovascular Science, Liverpool, Merseyside - Reino Unido da Grã-Bretanha; 9 Liverpool Reino Unido da Grã-Bretanha Liverpool Heart & Chest Hospital, Liverpool - Reino Unido da Grã-Bretanha; 10 Aalborg Dinamarca Aalborg Universitet - Aalborg Thrombosis Research Unit, Aalborg - Dinamarca

**Keywords:** Atrial Fibrillation, Epidemiology, Electrocardiography/methods, Anticoagulants, Longitudinal Study, Aged, Stroke, Embolism

## Abstract

**Background:**

Atrial fibrillation or flutter (AFF) is the most common sustained cardiac arrhythmia. Limited data can be found on AFF epidemiology in South America.

**Objective:**

The present study sought to describe the clinical epidemiology of AFF and the use of stroke prevention medication in the Brazilian Longitudinal Study of Adult Health (ELSA-Brasil) baseline assessment.

**Methods:**

This study analyzed data from 13,260 ELSA-Brasil participants. AFF was defined according to ECG recording or by self-report. Logistic regression models were built to analyze factors associated with AFF. This study also analyzed if age and sex were associated with anticoagulant use for stroke prevention. Significance level was set at 5%.

**Results:**

Median age was 51 years and 7,213 (54.4%) participants were women. AFF was present in 333 (2.5%) participants. Increasing age (odds ratio [OR]:1.05; 95% confidence interval [95%CI]: 1.04–1.07), hypertension (OR:1.44; 95%CI: 1.14–1.81), coronary heart disease (OR: 5.11; 95%CI: 3.85–6.79), heart failure (OR:7.37; 95%CI: 5.00–10.87), and rheumatic fever (OR:3.38; 95%CI: 2.28–5.02) were associated with AFF. From 185 participants with AFF and a CHA2DS2-VASc score ≥2, only 20 (10.8%) used anticoagulants (50.0% among those with AFF in the baseline ECG). Stroke prevention in this group was associated with a higher age (1.8% vs 17.7% in those aged ≤ 54 and ≥ 65 years, respectively; p=0.013). A trend towards a reduced anticoagulant use was observed in women (7.1% vs. 16.4% in women and men, respectively; p=0.055).

**Conclusions:**

At the ELSA-Brasil baseline, 2.5% of the participants had AFF. The lack of stroke prevention was common, which is an especially challenging point for healthcare in this setting.

## Introduction

Atrial fibrillation is the most prevalent sustained cardiac arrythmia, with an approximate lifetime risk of 25%^1^ and an estimated 33.3 million prevalent cases globally.^2^ In São Paulo, Brazil, a population-based study^3^ found that 3.2% of men and 2.0% of women aged 65 years or older had atrial fibrillation. Another large database of primary care patients aged 5 years or older (71% aged ≥ 40 years) in Minas Gerais, Brazil,^4^ found that 2.4% of men and 1.3% of women had atrial fibrillation. Risk factors for atrial fibrillation or flutter include age, hypertension, diabetes, smoking, obesity, heart failure, valve disease, and myocardial infarction.^5^^-^^7^

Risk-based thromboembolic (including stroke) prevention therapy is recommended for patients with atrial fibrillation or flutter (AFF).^8^^,^^9^ The CHA_2_DS_2_-VASc scores^10^ are currently the main strategy to evaluate thromboembolic risk in these individuals.^11^ Oral antithrombotic therapy is recommended for those with a CHA_2_DS_2_-VASc score ≥ 2.^9^

Oral anticoagulation therapy, however, still represents a challenge in clinical practice,^12^^,^^13^ and a significant proportion of AFF disease burden is still associated with incident stroke,^14^ especially in women.^15^^,^^16^ This is even more important in Brazil, where although age-adjusted incident rates of stroke in the country have been falling in recent years,^17^ stroke mortality is still high when compared to other South American countries, with a higher impact on the less developed states of the nation.^17^^,^^18^

The Brazilian Longitudinal Study of Adult Health (ELSA-Brasil) is a large multicenter cohort of individuals of 35 to 74 years of age in six Brazilian cities, whose aim is to study the incidence and associated factors for cardiovascular diseases and diabetes. The objectives of the present study are to describe the frequency of AFF at the ELSA-Brasil baseline and the use of stroke prevention medication in participants with AFF and a CHA_2_DS_2_-VASc score ≥ 2.

## Methods

### Study setting and design

The ELSA-Brasil design^19^ and cohort profile^20^ are detailed elsewhere. Briefly, ELSA-Brasil is a cohort study of 15,105 civil servants from six Brazilian cities (São Paulo, Belo Horizonte, Porto Alegre, Salvador, Rio de Janeiro, and Vitoria). Baseline assessments took place from August 2008 to December 2010 and included in-person interviews conducted by trained personnel, using clinical, laboratory, and imaging exams. Since baseline, all participants receive a yearly telephone follow-up contact. Four years after enrollment (2012-2014), all participants were invited to undergo onsite reassessment, which included new questionnaires, and clinical and laboratory evaluations. This reassessment was attended by 14,014 (92.8%) participants. Informed consent was obtained from all participants. Study protocol is in accordance to the Brazilian National Health Council 466/2012 resolution and was approved by the Institutional Review Board in each participating center.

### Atrial fibrillation or flutter diagnosis

Methods to record electrocardiographic (ECG) tracings in ELSA-Brasil are detailed elsewhere.^21^ ECG at the baseline was performed in each center, using a Burdick Atria 6100 device, calibrated at 10 mm/mV and a speed of 25 mm/second. The recordings were transmitted to the reading center in Minas Gerais Investigation Center. Analyses followed the Glasgow system,^22^ and were coded according to the Minnesota Coding System. Selected codes (including AFF) were manually reviewed by trained staff. In addition, in the onsite reassessment conducted in 2012-2014, participants were asked the following question: *“Did a physician ever say that you have/had atrial fibrillation?”* Participants who answered “yes” to that question were asked *“How old were you the first time a physician told you that you have/had atrial fibrillation?”*

The present study defined AFF diagnosis at the baseline if the participant (a) had an ECG recording with AFF in the ELSA-Brasil baseline assessment (n=48) or (b) indicated that they have had an atrial fibrillation diagnosis at an age lower than his/her age upon ELSA-Brasil enrollment (n=285). Most analyses are presented for all AFF cases and according to each definition criterion.

### Study sample

This study included ELSA-Brasil participants for whom AFF diagnosis at the baseline could be assessed. From the 15,105 ELSA-Brasil participants, 663 (4.4%) participants were excluded because they did not have a valid ECG reading at the baseline nor a valid answer for the AFF in the 2012-2014 onsite reassessment, and 1,182 (7.8%) were excluded because they had a valid ECG reading at the baseline without AFF, but without a valid answer for the AFF in the 2012-2014 onsite reassessment (impairing the assessment of paroxysmal AFF). Therefore, our main sample consisted of 13,260 (87.8%) participants.

### Other variables

Age, sex, race, level of education, monthly family income, and smoking status were self-reported and stratified accordingly. Monthly income was transformed into US dollars (using a conversion rate of BRL 2.00=USD 1.00, based on the exchange rate at the baseline). Race was defined, as adopted by the Brazilian National Census, as Black, Mixed, White, Asian, and Native (Indigenous). The anthropometric measurements in the ELSA-Brasil were assessed using standard techniques,^23^ and body mass index (BMI) was calculated by dividing weight by height squared. BMI was categorized as normal (< 25 kg/m^2^), overweight (≥ 25 kg/m^2^ and < 30 kg/m^2^), and obese (≥ 30 kg/m^2^). Hypertension was defined as the report of the use of medications to treat hypertension, a systolic blood pressure ≥ 140 mmHg, or a diastolic blood pressure ≥ 90 mmHg upon ELSA-Brasil baseline assessment. Diabetes was defined as a medical history of diabetes mellitus, the report of the use of medications to treat diabetes mellitus, a fasting serum glucose ≥ 126 mg/dl, HbA1c levels ≥ 6.5%, or a 2-h oral glucose tolerance test ≥ 200 mg/dl. Dyslipidemia was defined as the report of the use of lipid lowering treatment or an LDL cholesterol level ≥ 130 mg/dl. Abdominal obesity was defined as a waist circumference > 88 cm in women and > 102 cm in men. Previous medical diagnosis of heart failure, stroke, rheumatic fever, and thromboembolic event were assessed by self-report. Coronary artery disease was defined by a previous medical diagnosis of myocardial infarction or angina pectoris or by previous coronary revascularization. Ankle and brachial pressure measurements for ankle-brachial index (ABI) calculation is described in detail in Miname et al.^24^. According to the findings of that study, ABI was calculated as the ratio between the minimum leg systolic pressure and the maximum arm systolic pressure for higher sensitivity. Peripheral artery disease was defined as an ABI < 1.0.^24^^,^^25^ For medication use data, other than questionnaires, patients were asked to bring to the baseline assessment of all medications and prescription drugs they were currently taking.^26^ Use of anticoagulants was defined by using a medication under Anatomical Therapeutic Chemical (ATC) codes B01AA or B01AB. Use of antiplatelet agents was defined by using a medication under ATC code B01AC. For participants without previous coronary artery disease or stroke, 10-year atherosclerotic cardiovascular disease (ASCVD) risk was calculated according to the 2013 ACC/AHA Guideline on the Assessment of Cardiovascular Risk.^27^ CHA_2_DS_2_-VASc scores were calculated according to Lip et al.^11^ and categorized as 0 or 1 point, 2 or 3 points, and ≥ 4 points.

### Statistical analysis

Categorical variables are presented as counts and proportions, and were compared using Chi-squared or Fisher's exact tests. Continuous variables (age, body-mass index, ankle-brachial index and 10-year ASCVD risk) have non-normal distribution (p<0.001 for all, using the Anderson-Darling test), and are presented as medians and interquartile ranges and compared among groups using Mann-Whitney U tests. Crude and age- and sex-adjusted logistic regression models were built to analyze if AFF diagnosis at the ELSA-Brasil baseline was associated with age, sex, race, level of education, monthly income, smoking status, hypertension, diabetes and dyslipidemia diagnoses, BMI, abdominal obesity, peripheral artery disease, coronary heart disease, heart failure, stroke, rheumatic fever, and a 10-year CHD risk > 10%. Binary logistic regression was used to analyze the association with all AFF cases, while multinomial logistic regression was used to analyze the association with AFF cases according to each definition criterion (by ECG recording or self-report) in separate. The significance level was set at 5%, and R software, version 3.5.1, was used in all analyses.

## Results

Among the 13,260 participants included in the analyses, 333 (2.5%) had AFF at the ELSA-Brasil baseline, 176/7,213 (2.4%) women and 157/6,047 (2.6%) men. According to age strata, the frequency of AFF diagnosis was 1.2%, 2.2%, 2.9%, and 5.4% for ages < 45, 45-54, 55-64, and > 64 years, respectively. Table 1 shows the study's sample characteristics, according to the presence of AFF diagnosis at the ELSA-Brasil baseline. Individuals of non-white races represented 47.9% of the sample, and most participants had a college degree or higher education.

**Table 1:** Study sample characteristics according to the presence of atrial fibrillation or flutter in the ELSA-Brasil baseline

		No atrial fibrillation or flutter (N=12,927)	Atrial fibrillation or flutter				
			By ECG recording (N=48)	By self-report only (N=285)	All AFF cases (N=333)	Total (N=13,260)	p-value
Age (years; median [P25 - P75])		51.0 [45.0 – 58.0]	61.5 [56.0 – 71.0]	54.0 [49.0 – 62.0]	56.0 [49.0 – 63.0]	51.0 [45.0 – 58.0]	<0.001 ‡
Female sex (N (%))		7.037 (54.4%)	18 (37.5%)	158 (55.4%)	176 (52.9%)	7.213 (54.4%)	0.61 †
**Race (N (%))**							
	White	6.652 (52.1%)	29 (61.7%)	154 (54.6%)	183 (55.6%)	6.835 (52.1%)	0.039 †
	Mixed	3.608 (28.2%)	11 (23.4%)	74 (26.2%)	85 (25.8%)	3.693 (28.2%)	
	Black	2.069 (16.2%)	7 (14.9%)	35 (12.4%)	42 (12.8%)	2.111 (16.1%)	
	Other	449 (3.5%)	0 (0.0%)	19 (6.7%)	19 (5.8%)	468 (3.6%)	
**Level of education (N (%))**							
	Up to incomplete high school	1.529 (11.8%)	13 (27.1%)	34 (11.9%)	47 (14.1%)	1.576 (11.9%)	0.10 †
	High school	4.443 (34.4%)	12 (25.0%)	85 (29.8%)	97 (29.1%)	4.540 (34.2%)	
	College or above	6.955 (53.8%)	23 (47.9%)	166 (58.2%)	189 (56.8%)	7.144 (53.9%)	
**Monthly income (N (%))**							
	<USD1245	3.357 (26.1%)	15 (31.2%)	58 (20.4%)	73 (22.0%)	3.430 (26.0%)	0.001 †
	USD1245-3319	5.679 (44.1%)	12 (25.0%)	115 (40.5%)	127 (38.3%)	5.806 (43.9%)	
	≥ USD3320	3.845 (29.9%)	21 (43.8%)	111 (39.1%)	132 (39.8%)	3.977 (30.1%)	
**Smoking status (N (%))**							
	Never smoked	7.467 (57.8%)	29 (60.4%)	161 (56.5%)	190 (57.1%)	7.657 (57.7%)	0.75 †
	Past smoker	3.822 (29.6%)	13 (27.1%)	91 (31.9%)	104 (31.2%)	3.926 (29.6%)	
	Current smoker	1.637 (12.7%)	6 (12.5%)	33 (11.6%)	39 (11.7%)	1.676 (12.6%)	
Hypertension (N (%))		4.463 (34.5%)	30 (62.5%)	136 (47.7%)	166 (49.8%)	4.629 (34.9%)	<0.001 †
Diabetes (N (%))		2.438 (18.9%)	15 (31.2%)	66 (23.2%)	81 (24.3%)	2.519 (19.0%)	0.015 †
Dyslipidemia (N (%))		7.489 (58.0%)	27 (56.2%)	188 (66.0%)	215 (64.6%)	7.704 (58.1%)	0.019 †
Body-mass index (kg/m2; median [P25 - P75])		26.3 [23.7 – 29.5]	27.4 [25.4 – 30.4]	26.2 [23.9 – 30.2]	26.5 [24.1 – 30.3]	26.3 [23.7 – 29.6]	0.35 ‡
**Body-mass index classification (N (%))**							
	Normal	4.791 (37.1%)	10 (20.8%)	104 (36.5%)	114 (34.2%)	4.905 (37.0%)	0.057 †
	Overweight	5.238 (40.5%)	24 (50.0%)	102 (35.8%)	126 (37.8%)	5.364 (40.5%)	
	Obese	2.892 (22.4%)	14 (29.2%)	79 (27.7%)	93 (27.9%)	2.985 (22.5%)	
Abdominal obesity (N (%))		4.656 (36.0%)	20 (41.7%)	113 (39.6%)	133 (39.9%)	4.789 (36.1%)	0.16 †
Ankle-brachial index (median [P25 - P75])		1.18 [1.12 – 1.23]	1.13 [1.04 – 1.20]	1.17 [1.11 – 1.24]	1.16 [1.10 – 1.23]	1.18 [1.12 – 1.23]	0.008 ‡
Peripheral artery disease (N (%))		403 (3.1%)	6 (12.5%)	12 (4.2%)	18 (5.4%)	421 (3.2%)	0.025 †
Use of anticoagulants (N (%))		37 (0.3%)	19 (39.6%)	5 (1.8%)	24 (7.2%)	61 (0.5%)	<0.001 †
Use of antiplatelet agents (N (%))		706 (5.5%)	7 (14.6%)	45 (15.8%)	52 (15.6%)	758 (5.7%)	<0.001 †
Use of anticoagulant and/or antiplatelet agents (N (%))		741 (5.7%)	26 (54.2%)	50 (17.5%)	76 (22.8%)	817 (6.2%)	<0.001 †
Coronary artery disease (N (%))		536 (4.2%)	7 (14.6%)	67 (23.5%)	74 (22.2%)	610 (4.6%)	<0.001 †
Heart failure (N (%))		160 (1.2%)	10 (20.8%)	26 (9.2%)	36 (10.8%)	196 (1.5%)	<0.001 †
Stroke (N (%))		153 (1.2%)	1 (2.1%)	7 (2.5%)	8 (2.4%)	161 (1.2%)	0.067 †
Rheumatic fever (N (%))		352 (2.7%)	7 (14.6%)	23 (8.1%)	30 (9.0%)	382 (2.9%)	<0.001 †
Thromboembolic event (N (%))		198 (1.5%)	4 (8.3%)	5 (1.8%)	9 (2.7%)	207 (1.6%)	0.11 †
10-year ASCVD risk (median [P25 - P75])		2.8% [1.1% −7.1%]	11.8% [4.2% – 18.5%]	2.7% [1.4% – 7.9%]	3.4% [1.5% - 10.4%]	2.8% [1.1% – 7.2%]	0.001 ‡
10-year ASCVD risk > 10% (N (%))		2075 (16.9%)	23 (57.5%)	41 (19.2%)	64 (25.2%)	2139 (17.1%)	0.001 †

The 10-year ASCVD risk is defined only in participants without prior coronary artery disease or stroke. AFF: Atrial fibrillation or flutter. ASCVD: Atherosclerotic cardiovascular disease. P-values are presented for the comparison between the no atrial fibrillation or flutter (N=12,927) and all AFF case (N=333) groups, using

† Chi-squared or

‡ Mann-Whitney U tests

Table 2 shows crude and age- and sex-adjusted odds ratios for the association with the presence of AFF diagnosis at the ELSA-Brasil baseline. Considering all AFF cases, higher age (p<0.001), higher income (p=0.044), hypertension (p=0.002), coronary heart disease (p<0.001), heart failure (p<0.001), and rheumatic fever diagnoses (p<0.001) were positively associated with AFF in the adjusted models. Restricting the cases to the 48 participants who presented AFF in the baseline ECG, higher age (p<0.001), male sex (p=0.037), peripheral artery disease (p=0.026), heart failure (p<0.001), and rheumatic fever diagnoses (p<0.001) were positively associated with AFF in the adjusted models. Supplemental Table 1 shows crude model results for these associations.

**Table 2:** Age- and sex-adjusted odds ratios (95% CIs) for the association with atrial fibrillation or flutter in the ELSA-Brasil baseline

			Atrial fibrillation or flutter	
Variable		By ECG recording (N=48)	By self-report only (N=285)	All AFF cases (N=333)
Age (one-year increase)		1.12 [1.09 – 1.16]	1.04 (1.03 – 1.06) †	1.05 (1.04 – 1.07) †
Female sex		0.53 (0.30 – 0.96) †	1.05 (0.83 – 1.33)	0.95 (0.76 – 1.18)
**Race** *				
	White	1.0 (Reference)	1.0 (Reference)	1.0 (Reference)
	Mixed	0.92 (0.45 – 1.85)	0.95 (0.72 – 1.26)	0.94 (0.72 – 1.22)
	Black	(0.43 – 2.27)	0.76 (0.52 – 1.10)	0.79 (0.56 – 1.10)
**Level of education**				
	Up to incomplete high school	1.73 (0.87 – 3.43)	0.79 (0.54 – 1.15)	0.92 (0.66 – 1.27)
	High school	1.12 (0.55 – 2.28)	0.86 (0.66 – 1.12)	0.88 (0.69 – 1.13)
	College or above	1.0 (Reference)	1.0 (Reference)	1.0 (Reference)
**Monthly income**				
	<USD1245	1.27 (0.64 – 2.51)	0.67 (0.48 – 0.93) †	0.74 (0.55 – 0.99) †
	USD1245-3319	0.61 (0.29 – 1.25)	0.80 (0.61 – 1.04)	0.77 (0.60 – 0.99) †
	≥ USD3320 (N (%))	1.0 (Reference)	1.0 (Reference)	1.0 (Reference)
**Smoking status**				
	Never smoked	(Reference)	(Reference)	1.0 (Reference)
	Past smoker	0.57 (0.29 - 1.12)	0.98 (0.75 – 1.27)	0.90 (0.70 – 1.16)
	Current smoker	1.00 (0.42 – 2.40)	0.93 (0.64 – 1.36)	0.93 (0.65 – 1.32)
Hypertension		1.65 (0.89 – 3.03)	1.41 (1.10 – 1.81) †	1.44 (1.14 – 1.81) †
Diabetes		1.11 (0.60 – 2.08)	1.06 (0.80 – 1.41)	1.07 (0.82 – 1.39)
Dyslipidemia		0.66 (0.37 – 1.18)	1.22 (0.95 – 1.57)	1.10 (0.87 – 1.39)
**Body-mass index classification**				
	Normal	1.0 (Reference)	1.0 (Reference)	1.0 (Reference)
	Overweight	1.88 (0.90 – 3.95)	0.85 (0.64 – 1.12)	0.94 (0.72 – 1.21)
	Obese	2.21 (0.97 – 5.00)	1.19 (0.88 – 1.60)	1.27 (0.96 – 1.68)
Abdominal obesity		1.15 (0.63 – 2.08)	1.04 (0.81 – 1.33)	1.05 (0.83 – 1.32)
Peripheral artery disease		2.72 (1.13 – 6.54) †	1.16 (0.64 – 2.10)	1.44 (0.88 – 2.35)
Coronary artery disease		1.87 (0.81 – 4.28)	5.99 (4.44 – 8.09) †	5.11 (3.85 – 6.79) †
Heart failure		11.67 (5.58 – 24.40) †	6.51 (4.19 – 10.11) †	7.37 (5.00 – 10.87) †
Stroke		0.90 (0.12 – 6.67)	1.65 (0.76 – 3.57)	1.51 (0.73 – 3.13)
Rheumatic fever		5.75 (2.55 – 12.98) †	3.02 (1.94 – 4.70) †	3.38 (2.28 – 5.02) †
10-year ASCVD risk > 10%		1.56 (0.63 – 3.85)	0.78 (0.50 – 1.21)	0.95 (0.65 – 1.40)

* The small proportion of individuals of other races in the sample precluded estimation. AFF: Atrial fibrillation or flutter. ASCVD: Atherosclerotic cardiovascular disease. Odds ratios and p-values were obtained from logistic regression models adjusted for age and sex.

† p<0.05.

CHA_2_DS_2_-VASc scores could be calculated for 331 (99.4%) of the 333 participants with AFF diagnosis at the baseline (Table 3). As expected, individuals with higher CHA_2_DS_2_-VASc scores were older (p<0.001), were more likely to be women (p=0.002), and presented a higher cardiovascular risk (p<0.001). Among 185 participants with a CHA_2_DS_2_-VASc score ≥ 2, only 20 (10.8%) received anticoagulant therapy (16/32; 50.0% when restricting cases to the 48 participants who presented AFF in the baseline ECG).

**Table 3:** Characteristics of participants with atrial fibrillation or flutter in the ELSA-Brasil baseline according to CHA_2_DS_2_-VASc scores

	Atrial fibrillation or flutter						
	By ECG recording		By self-report only		All AFF cases		
	CHA_2_DS_2_-VASc score < 2 (N=16)	CHA_2_DS_2_-VASc score ≥ 2 (N=32)	CHA_2_DS_2_-VASc score < 2 (N=130)	CHA_2_DS_2_-VASc score ≥ 2 (N=153)	CHA_2_DS_2_-VASc score < 2 (N=146)	CHA_2_DS_2_-VASc score ≥ 2 (N=185)	p-value
Age (years; median [P25 - P75])	56.0 [49.5 – 61.2]	67.5 [58.0 – 71.2]	49.5 [45.0 – 56.0]	59.0 [53.0 – 66.0]	50.0 [45.0 – 57.0]	60.0 [53.0 – 68.0]	<0.001 ‡
Female sex (N (%))	3 (18.8%)	15 (46.9%)	60 (46.2%)	97 (63.4%)	63 (43.2%)	112 (60.5%)	0.002 †
Coronary artery disease (N (%))	0 (0.0%)	7 (21.9%)	2 (1.5%)	64 (41.8%)	2 (1.4%)	71 (38.4%)	<0.001 †
Stroke (N (%))	0 (0.0%)	1 (3.1%)	0 (0.0%)	7 (4.6%)	0 (0.0%)	8 (4.3%)	0.010 ¥
10-year ASCVD risk > 10% (N (%))	7 (43.8%)	16 (66.7%)	15 (11.7%)	26 (30.6%)	22 (15.3%)	42 (38.5%)	<0.001 †
Prior ASCVD or 10-year ASCVD risk > 10%	7 (43.8%)	24 (75.0%)	17 (13.1%)	94 (61.4%)	24 (16.4%)	118 (63.8%)	<0.001 †
Use of anticoagulants (N (%))	3 (18.8%)	16 (50.0%)	1 (0.8%)	4 (2.6%)	4 (2.7%)	20 (10.8%)	0.005 ¥
Use of antiplatelet agents (N (%))	2 (12.5%)	5 (15.6%)	2 (1.5%)	43 (28.1%)	4 (2.7%)	48 (25.9%)	<0.001 †

The 10-year ASCVD risk is defined only in participants without prior coronary artery disease or stroke. AFF: Atrial fibrillation or flutter. ASCVD: Atherosclerotic cardiovascular disease. P-values are presented for the comparison between the CHA2DS2-VASc score < 2 (N=146) and the CHA2DS2-VASc score ≥ 2 (N=185) groups among all AFF cases using

† Chi-squared,

‡ Mann-Whitney U or

¥ Fisher's exact tests

Table 4 shows the prevalence of oral anticoagulant use in participants with AFF and a CHA_2_DS_2_-VASc score ≥ 2, according to age and sex. Considering all AFF cases, anticoagulant use was more frequent in those with a higher age (p=0.013), and a trend towards lower anticoagulant use in women (p=0.055). Supplemental table 2 shows that the frequency of anticoagulant use in men was numerically higher than in women for all age strata, but no significant differences were discovered when analyzing all AFF cases nor when restricting them to those participants who presented with AFF in the baseline ECG.

**Table 4:** Frequency of anticoagulant use according to age and sex in participants with atrial fibrillation or flutter and a CHA_2_DS_2_-VASc score ≥ 2 in the ELSA-Brasil baseline

			Atrial fibrillation or flutter and a CHA_2_DS_2_-VASc score ≥ 2		
Variable		By ECG recording (N=32)	By self-report only (N=153)	All AFF cases (N=185)	p-value
**Age (years)**					0.013
	≤ 54	1 / 3 (33.3%)	0 / 52 (0.0%)	1 / 55 (1.8%)	
	55 – 64	6 / 11 (54.5%)	2 / 57 (3.5%)	8 / 68 (11.8%)	
	≥ 65	9 / 18 (50.0%)	2 / 44 (4.5%)	11 / 62 (17.7%)	
**Sex**					0.055
Male	9 / 17 (52.9%)	3 / 56 (5.4%)	12 / 73 (16.4%)		
	Female	7 / 15 (46.7%)	1 / 97 (1.0%)	8 / 112 (7.1%)	

The frequency of anticoagulant use according to age and sex, considering all AFF cases with a CHA_2_DS_2_-VASc score ≥ 2 was compared using Fisher's exact tests.

## Discussion

### Contextualization and discussion of main findings

In our cohort sample, 2.5% of the included participants presented AFF at the baseline. Age, male sex, income, hypertension, coronary heart disease, peripheral artery disease, heart failure, and rheumatic fever diagnoses were positively associated with AFF. Second, most participants with AFF and a CHA_2_DS_2_-VASc score ≥ 2 did not receive anticoagulants and/or antiplatelet agents. Third, oral anticoagulant use was more common in those with a higher age, while women were less commonly treated.

Comparing AFF prevalence across samples is challenging because of differences in study design, study setting, and age and sex distribution upon recruitment. A review of the global epidemiology of atrial fibrillation^28^ found a reported worldwide prevalence ranging from 0.1% to 6.0%, depending on the age strata and sex analyzed. Although this study reports a similar frequency of AFF diagnosis in the sample when compared to the cited Brazilian studies,^3^^,^^4^ these studies have marked differences and are complementary. For example, Kawabata-Yoshihara et al.^3^ performed a systematic door knock of individuals 65 years of age or older, while Marcolino et al.^4^ used a large ECG database from the Telehealth Network of Minas Gerais consisting of individuals 5 years of age or older who received medical care in primary care units. Both studies considered AFF diagnosis according to ECG recordings upon assessment. When comparing the findings from those studies to the findings from the present study, a higher frequency of AFF was found when similar age strata are compared. This is most likely influenced by case definitions, as the present study also included a self-reported medical diagnosis as a diagnostic criterion. However, considering only the ECG tracings during assessment and excluding medical history from case definitions may yield the highest specificity and reduce self-reported information error, but it can also underestimate prevalence rates due to the under-recognition of paroxysmal atrial fibrillation.

The association between heart failure and atrial fibrillation has clinical importance. Healey et al.^12^ analyzed data from 15,400 individuals with AFF in 47 countries. After one year of follow-up, death occurred in 11% of the cohort, and heart failure was the most common cause of death (30%). Similarly, individuals with peripheral artery disease and AFF have a higher risk of stroke when compared to those with only AFF.^29^ It is important to note that, in recent decades, rheumatic heart disease has decreased as a cause of mortality in Brazil,^30^ and although new cases in the country are less common, current middle-aged and older adults may have had rheumatic fever during childhood. Therefore, the prevalence and mortality due to rheumatic heart disease in Brazil is not negligible,^31^ which is reinforced by our finding that 9.0% of the individuals with AFF in this study's sample (and 2.9% overall) presented a medical history of rheumatic fever.

A striking finding of the present study was that 89.2% of all participants with AFF and a CHA_2_DS_2_-VASc score ≥ 2 did not receive anticoagulants. This is more important considering that ELSA-Brasil participants have a higher mean level of education and income when compared to the general Brazilian population.^20^ Although self-reported diagnoses may have influenced our finding of low rates of stroke prevention, when we limited analyses to participants with a documented AFF in the baseline ECG recording, half of them still had not received anticoagulants.

Although concerning, these low stroke prevention prescription rates are not exclusive to our sample. A similar study conducted by Healey et al.^12^ described that, by geographic region, 32% (North America, Western Europe, Australia, and the Middle East) to 70% (China) guideline-indicated patients did not receive oral anticoagulant therapy. In South America, where ELSA-Brasil is located, the frequency of non-prescription was 55%. Ogilvie et al.^32^ reported a systematic review of studies reporting the use of oral anticoagulants in atrial fibrillation outside of clinical trials. They found that 21/29 (72.4%) studies in patients with prior stroke or transient ischemic attack, as well as 5/9 (55.6%) studies in patients with high thromboembolic risk according to risk scores, reported prescriptions levels of below 60%.

The under-prescription of stroke prevention medication seems to be a more severe problem in women,^33^ which is in accordance with our data. Khurshid et al.^34^ analyzed electronic medical data from 4,388 patients with atrial fibrillation and found that women, when compared to men, received less anticoagulant therapy at 1, 3, and 6 months after atrial fibrillation diagnosis. In addition, Emdin et al.^35^ performed a systematic review and meta-analysis of 30 cohort studies and found that women, as compared to men, with atrial fibrillation had a higher risk of all-cause mortality, cardiovascular mortality, and stroke. These authors highlight that, besides the underuse of stroke prevention medication, other explanations for worse outcomes, such as the higher rates of adverse effects from anticoagulant^36^ and antiarrhythmic^37^ therapies in women are equally plausible. In addition, some authors have highlighted worse anticoagulation control in women receiving warfarin. For example, Sullivan et al.^38^ analyzed data from 4,060 participants of the Atrial Fibrillation Follow-up Investigation of Rhythm Management (AFFIRM) trial, and found that women, as compared to men, on warfarin spent significantly more time below the international normalized ratio (INR) therapeutic range (29% vs 26%). It is also important to note that, the ELSA-Brasil baseline assessment occurred from 2008 to 2010. At that time, the use of direct-acting oral anticoagulants for stroke prevention in AFF was very rare.

### Strengths and limitations

This study does have some strengths. We analyzed data from a large, multicenter sample of individuals who were not recruited from clinical settings. This reduces potential biases in the study of factors associated with atrial fibrillation and diagnosis and the prescription of stroke prevention medications, approximating our external validity to the general population. The comprehensive ELSA-Brasil protocol allowed for the analysis of medication use according to CHA_2_DS_2_-VASc thromboembolic risk scores. This study must also be interpreted within its context. As stated earlier, this study's case definition may be prone to self-reported information misclassifications. Although this strategy minimizes the under-recognition of paroxysmal atrial fibrillation, it is important to acknowledge that self-reported cases were an important proportion of all AFF cases, and this may have influenced some of our results. Data on the frequency of individuals within the INR therapeutic range or on bleeding risk scores was not available. However, we believe that the risk of high bleeding is unable to explain a substantial part of the lack of stroke prevention in this sample.

## Conclusions

In conclusion, a 2.5% frequency of AFF diagnosis was found at the ELSA-Brasil baseline assessment. Age, male sex, peripheral artery disease, heart failure, or rheumatic fever diagnoses were associated with AFF. Almost 90% of the subsample of participants with AFF and a CHA_2_DS_2_-VASc score ≥ 2 (50% considering only those diagnosed by ECG records) did not receive anticoagulants.
